# Radon exposure and risk of cerebrovascular disease: a systematic review and meta-analysis in occupational and general population studies

**DOI:** 10.1007/s11356-022-20241-x

**Published:** 2022-04-23

**Authors:** Liping Lu, Yijia Zhang, Cheng Chen, Robert William Field, Ka Kahe

**Affiliations:** 1 Department of Obstetrics and Gynecology and Department of Epidemiology, Columbia University Irving Medical Center, 622 West 168th Street, New York, NY 10032 USA; 2 Department of Occupational and Environmental Health and Department of Epidemiology, College of Public Health, University of Iowa, Iowa City, IA 52242 USA

**Keywords:** Radon, Radon decay product, Cerebrovascular disease, Stroke, Occupational population, General population, Meta-analysis

## Abstract

Although it is biologically plausible, findings relating radon exposure to the risk of cerebrovascular disease (CeVD) are inconsistent and inconclusive. To investigate whether radon exposure was associated with the risk of CeVD, we qualitatively and quantitatively summarized the literature on radon and CeVD in both occupational and general populations. A search of PubMed, Embase, Scopus, and Web of Science was performed for peer-reviewed articles published through March 2022. Studies were excluded if radon exposure was not assessed separately from other ionizing radiation. In the meta-analysis, excess relative risks (ERRs) were converted to relative risks (RRs), and the pooled RRs and 95% confidence intervals (CIs) were determined using the random-effects model (DerSimonian and Laird). In the systematic review, nine eligible studies were summarized. Six occupational studies indicated inconsistent associations between cumulative radon exposure and CeVD mortality among mine workers. With available data from four updated occupational studies (99,730 mine workers and 2745 deaths), the pooled RR of radon exposure with CeVD mortality showed a non-significant association (1.10, 95% CI 0.92, 1.31). Three studies (841,270 individuals and 24,288 events) conducted in general populations consistently demonstrated a significant inverse relationship between residential radon exposure and risk of CeVD. The existing literature suggested a potential link between radon exposure and CeVD risk in general population. The inconsistent association in occupationally exposed populations may be explained by different methods of radon assessment and other methodological issues. Since radon exposure is a common public health issue, more rigorously designed epidemiologic studies, especially in the general population are warranted.

**Supplementary Information:**

The online version contains supplementary material available at 10.1007/s11356-022-20241-x.

## Introduction

Cerebrovascular disease (CeVD), with stroke being its most common manifestation, is a health disorder of the blood vessels supplying the brain (Tong et al. 2019). Clinically, CeVD can be further categorized into ischemic and hemorrhagic disease (William 1996). CeVD remains a major cause of morbidity and mortality globally (Koton et al. 2014). In addition to lifestyle and genetic determinants, the importance of environmental factors for CeVD development has been increasingly recognized (Reis et al. 2018).

Radon-222 (hereafter called radon) is a colorless, odorless, and radioactive gas that occurs naturally in the environment. It is produced from the radioactive decay of uranium-238, and subsequently radium-226, and generally enters a house through cracks and fissures (WHO 2009). The radioactive decay of radon leads to the formation of a set of solid radon decay products (RDPs), delivering most of the radiologic dose by ionizing radiation of alpha particles. For the remainder of the paper, radon refers to both radon gas and RDPs.

Radon exposure is considered to be similar to tobacco smoking regarding its health impact on lung cancer (Alavanja 2002). Previous research has established the effect of radon exposure on lung cancer (Field et al. 2000); however, the potential mechanisms in relation to diseases other than cancer, including CeVD risk, have not been completely understood. During respiration, radon can dissolve into the bloodstream through gas exchange, enter systemic circulation, and diffuse to all tissues, particularly the liver and kidney (Peterman and Perkins 1988). There are multiple pathways that potentially link radon exposure to the development of CeVD: First, radon radiation induces the development of artheromas on the arterial wall through the accumulation of LDL particles and lipoprotein, the migration of smooth muscle cells, and the formation of foam cells and fibrous lesions (Johnson and Duport 2004). Fibrosis or thickening of arteries, resulting from radon radiation, can damage the blood vasculature (Robertson et al. 2013). Second, radon may stimulate inflammation that leads to endothelial damage and dysfunction, and consequently the development of atherosclerosis (Little et al. 2010). Third, radon induces the production of oxygen species (ROS) (Xin et al. 2022), which may lead to vascular injury, inflammatory reaction, and atherogenesis (Kattoor et al. 2017).

Although it is biologically plausible, the research has not focused on non-cancer outcomes until recent years. In particular, the studies on the association between radon and CeVD are sparse, and the findings are inconsistent and inconclusive. Therefore, we performed a systematic review and meta-analysis to qualitatively and quantitatively sum up the available epidemiologic evidence of CeVD outcomes associated with radon exposure.

## Methods and materials

The study implementation was guided by Preferred Reporting Items for Systematic Reviews and Meta-Analyses (PRISMA) checklist (Moher et al. 2009).

### Search strategy

A thorough search for relevant peer-reviewed publications was performed in four electronic databases (PubMed, Embase, Scopus, and Web of Science). Because radon and its decay products both deliver alpha emissions, exposure in this study consisted of both radon and RDPs. Also, CeVD includes stroke, transient ischemic attack, aneurysms, vascular malformations, vascular dementia, and subarachnoid hemorrhage (Portegies et al. 2016). Thus, the search terms included a combination of keywords including “radon” or “RDP” and “cerebrovascular disease,” “stroke”, “cardiovascular disease,” “transient ischemic attack,” “cerebral aneurysm,” “vascular malformation,” “vascular dementia,” or “subarachnoid hemorrhage.” The literature search was conducted from inception to March 2022. Detailed literature search strategies in electronic databases were reported in Supplemental Table 1.

### Study selection

Study eligibility is developed based on the PECO guideline consisting of “population, exposure, comparator, and outcome” (Supplemental Table 2). Studies were considered for the systematic review if they met the following criteria: (1) published in English; (2) original studies (i.e., cohort, case-control, or cross-sectional studies); (3) had radon or RDPs as the exposure; and (4) had morbidity/mortality of CeVD/stroke reported by relative risk (RR), hazard ratio (HR), odds ratio (OR), standard mortality rate (SMR), or excess relative risk (ERR). We excluded the studies if (1) they were not human studies and (2) radon exposure was not assessed separately from other sources of ionizing radiation. In case of multiple studies reporting the same results of interest based on the same cohort, only the most recent publications including the information derived from the longest follow-up were included. Studies reported with ERR and corresponding 95% CI were included into meta-analysis.

Literature search results were imported to a text file. The first reviewer (LL) screened all titles and abstracts. The second reviewer (YZ) participated in further full text inspection. Discrepancies that arose following full-text screening were resolved through discussion between the two reviewers. None of the reviewers had conflicts of interest with the relevant publications that would be included in this study.

### Data collection

For each primary study identified, we extracted the following information: lead author, publication year, study characteristics (e.g*.*, region, study design, population, and sample size), participants’ average age at study entry, follow-up years, exposure measurement, outcomes confirmation, exposure doses, risk estimate and the corresponding 95% confidence intervals (CIs), and adjusted covariates in the final model. LL collected the data from the primary studies, and YZ provided an independent review for data accuracy.

### Quality assessment

Based on the risk of bias (ROB) rating tool for human and animal studies developed by the National Toxicology Program Office of Health Assessment and Translation (NTP/OHAT) (National Toxicology Program 2015), two reviewers (LL and YZ) assessed the ROB for each primary study separately. The quality of each study was rated based on seven probing questions that cover six possible domains of bias: selection, confounding, attrition/exclusion, detection, selective reporting, and other sources of bias. Except for detection bias, which consists of two questions, all other domains include one relevant question each. The scoring for each question is “definitely low,” “probably low,” “probably high,” or “definitely high.” According to the recommendation by OHAT, we selected three questions (one for confounding bias and two for detection bias) as the key questions. The overall quality was measured using 3-Tier approach (National Toxicology Program 2019): Tier 1 for high quality, Tier 2 for moderate quality, and Tier 3 for low quality. A summary of the study quality using the 3-Tier approach was presented in Supplemental Table 3. A third reviewer (KK) reconciled the disagreements.

### Statistical analysis

Some primary studies reported ERR, which was calculated using the linear model $RR\left(t,w\right)=1+\beta \times w\left(t\right)$, where $\beta$ estimates ERR for each unit of radon exposure and *w*(*t*) is the cumulative radon exposure at time *t*. ERR was estimated using an internal Poisson regression model with non-exposed individuals as the internal control. For example, ERR per 100 working level months (WLM) represented the increased risk of CeVD mortality with 100 WLM radon exposure relative to the baseline CeVD mortality. Because statistical methods of combining weighted ERR have not been well established, we converted ERR back to RR for the meta-analysis using the formula $\mathrm{RR}=\mathrm{ERR}+1$ (Mould 1998).

We estimated the pooled RRs (95% CI) using the DerSimonian and Laird random-effects model (DerSimonian and Laird 2015). We evaluated the heterogeneity using Cochran’s *Q* test and *I*^2^ statistics (in forest plots) (Higgins et al. 2003). A leave-one-out sensitivity analysis was performed to determine the impact of any individual study on the pooled estimate by excluding each single study at a time. Egger’s regression test was conducted to evaluate publication bias (Egger et al. 1997).

We used STATA software (version 16.0, STATA Corp., College Station, Texas) to conduct all of the analyses. A *p* value ≤ 0.05 was statistically significant.

## Results

### Study selection and characteristics

**Fig. 1:**
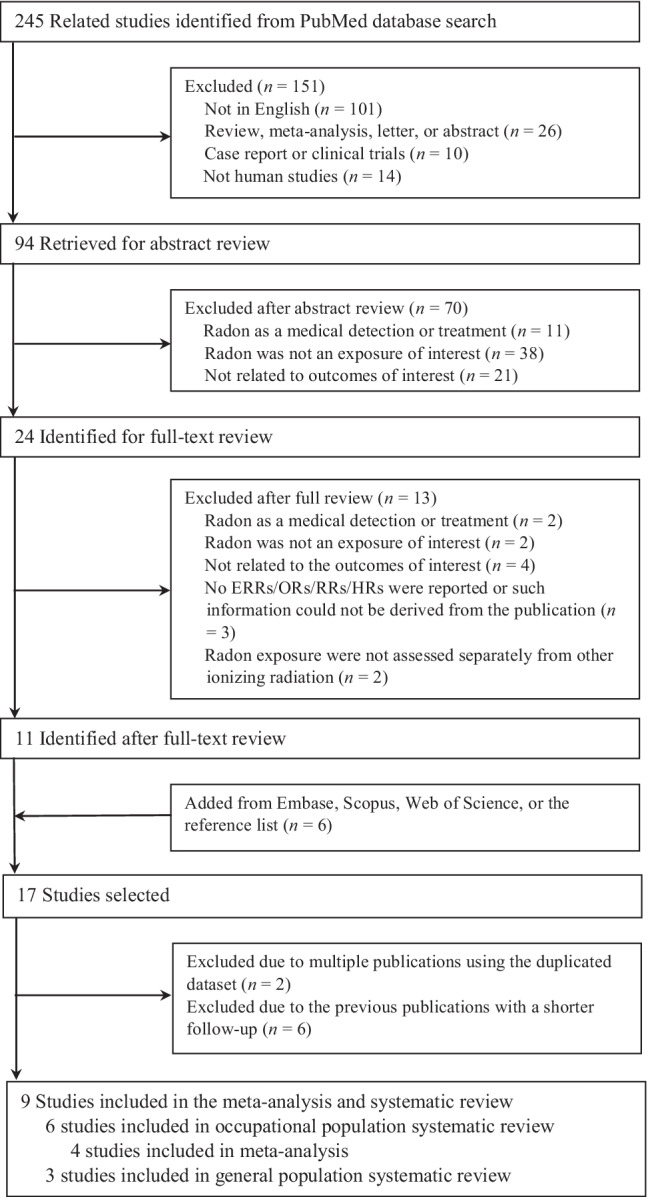
The process flowchart with the main steps of the literature screening and study selection

We screened 245 studies from PubMed during the first round of literature search, of which 24 publications were selected for full text review. We further excluded 11 studies for the reasons indicated in Fig. 1. In addition, we identified 6 studies from Embase, Scopus, Web of Science, or the reference lists of the published studies.

Among the 17 studies, 14 studies (7 distinctive cohorts) were conducted in occupational populations and 3 studies were conducted in the general population. Of the 14 occupational studies, we further excluded 8 studies because they were published previously with a shorter follow-up on the same cohort (Drubay et al. 2015; Kreuzer et al. 2006, 2010; Lane et al. 2010; Nusinovici et al. 2010; Rage et al. 2015) or using the duplicated database (Kreuzer et al. 2015; Zablotska et al. 2013). Thus, six occupational studies and three residential studies were summarized in the systematic review. Finally, four studies reporting ERR and corresponding 95% CI were included into meta-analysis (Kreuzer et al. 2013; Navaranjan et al. 2016; Rage et al. 2018; Zablotska et al. 2018) (Fig. 1).

### Study characteristics

These studies comprised data from cohort studies conducted in Canada (Navaranjan et al. 2016; Villeneuve et al. 2007), China (Xuan et al. 1993), France (Rage et al. 2018), Germany (Kreuzer et al. 2013), South Korea (Kim et al. 2020), and the USA (Klotz et al. 1989; Turner et al. 2012a). One study was a pooled analysis of Canadian and German mining workers without underground experience (Zablotska et al. 2018). The sample size of a single study ranged from 752 (Klotz et al. 1989) to 811,961 (Turner et al. 2012a). The length of follow-up ranged from 11 (Xuan et al. 1993) to 62 (Kreuzer et al. 2013) years (Tables 1 and 2).

**Table 1:** Characteristics of the six cohort studies in occupational population

Author (y)	Region	N of participants	Age^*a*^, y	Follow-up, y	Exposure assessment	Outcome confirmation	Exposure doses (WLM)	Risk estimates	Adjusted covariates
Rage et al. 2018^*b*^	France	5400 male uranium miners	29.0	1946–2007mean: 34.7	Experts retrospectively reconstruction (1946–1955); Individually assessed and recorded (1956–1982); ISID (from 1983)	RNIPP and CepiDC CeVD: ICD-7, ICD-8, ICD-9 (code 430–438), ICD-10 (code I60-I69)	35.1	*N*_CeVD death_ = 105 ERR/100 WLM 0.42 (0.04, 1.04) (*p* = 0.02)	Unadjusted
Xuan et al. 1993	China	17,143 male tin miners	18.3	1976–1987	Estimated from data on work history and on working level concentration	Through hospital and other records. Coded according to the Chinese Health Ministry coding system	275.4	*N*_CeVD death_ = 302 Age-adjusted RR by tertiles of randon exposure. RR: 1.0 (low) 1.1 (medium) 1.3 (high)	Age
Villeneuve et al. 2007	Canada	2070 Fluorspar workers with 1742 underground miners and 328 surface mill workers	NA	1950–2001	No monitoring and based on a variety of sources (before 1960; Taken from 700 samples annually (1961–1967) Daily radon exposures per worker were estimated based on levels measured in the place worked in a given day (from 1968)	CMDB. CeVD: ICD-9 (code 430–438)	348 Cumulative radon exposure (WLM): 0 > 0–100 > 100–400 > 400–800 > 800–1600 > 1600	*N*_CeVD death_ = 48 RR (95% CI) 1.0 (referent) 0.63 (0.30, 1.32) 0.73 (0.32, 1.66) 0.49 (0.18, 1.34) (*p* trend = 0.55)	Age, calendar period and lifetime smoking status (never, ever, unknown)
Navaranjan et al. 2016^*b*^	Canada	28,546 male uranium miners	28.8	1954–2007	Stationary area sampling	CMDB and CCDB CeVD: ICD-9 (code 430–438)	21.0	*N*_CeVD death_ = 315 ERR/100 WLM 0.22 (− 0.12, 0.58)	Age and calendar period
Kreuzer et al. 2013^*b*^	Germany	58,982 male uranium miners	25	1946–2008 mean:38	Experts estimation (before 1955) JEMs (from 1955)	Public Health Administrations and the pathology archive of the company. CeVD: ICD-10 (code I60–I69)	280	*N*_CeVD death_ = 2073 ERR/100WLM: 0.000 (− 0.008, 0.009)	Unadjusted
Zablotska et al., 2018^*b*^	Canada Germany	6802 male uranium workers with no mining experience (2641 in Canada and 4161 in Germany)	Canadian: 29 German: 30	Canadian: 1950–1999 German: 1946–2008 mean: Canadian:31 German: 23	Canadian: based on radium German: JEMs	Canadian: CMDB CeVD: ICD-9 (code 430–438) German: the Public Health offices and their archives and the autopsy files from company CeVD: ICD-10 (code I60–I69)	Total: 10.0 Canadian: 13.3 German: 8.5	*N*_stroke death_ = 252 ERR/100 WLM (95% CI): − 0.07 (< − 0.40, 0.52)	Calendar time, age at risk, cohort and duration of employment (< 6 vs*.* 6 + months; Port Hope cohort only) by stratification

*CCDB*, Canadian Cancer Data Base; *CepiDC*, the national Epidemiological Center on Medical Causes of Death; *CeVD,* cerebrovascular disease; *CI*, confidence interval; *CMDB*, Canadian Mortality Database; *ERR*, excess relative risk; *ICD*, International Classification of Diseases; *ISID*, integrated system of individual dosimetry; *JEM*, job-exposure matrix; *N*, number; *NA*, not available; *RNIPP*, the National Directory for the Identification of Natural Persons; *RR*, relative risk; *USA*, the United States of America; *WL*, working levels; *WLM*, working-level month; *Y*, year

^*a*^Mean age at employment, entry into study or first exposure to radon

^*b*^Included into meta-analysis

**Table 2:** Characteristics of the three studies in general population

Author (year)	Region	Study design	N of participants	Age^*a*^, y	Follow-up, y	Exposure assessment	Outcome confirmation	Exposure doses	Risk estimates	Adjusted covariates
Klotz et al. 1989	USA	Cohort	752	NA	1923–1983 mean: 25.2	Carbon canister detectors and alpha scintillation detectors	City directories, available local resources, New Jersey statistics and New Jersey motor vehicle records were used. A genealogy specialist served as consultant	0.078 WLM	*N*_CeVD death_ = 18 SMR (95% CI): 1.71 (1.01, 2.59) (compared with New Jersey mortality rates)	Unadjusted
Turner et al. 2012a, b	USA	Cohort	811,961	57	1982–2006	In LBL, indoor radon monitoring data along with other data were used to predict the annual average radon concentrations in 3079 counties; In Cohen, complied a series of screening measurements in a nonrandom sample of homes in 1601 counties	National Death Index. CeVD: ICD-9 (code 430–438); ICD-10 (code I60–I69)	53.5 Bq/m^3^	*N*_CeVD death_ = 23,344 HR/100 Bq/m^3^: 1.05 (0.99, 1.10) for LBL. HR/100 Bq/m^3^: 1.07 (1.01, 1.14) for Cohen’s data	Age, race, gender, and state stratified and adjusted for education, marital status, BMI, BMI-squared, cigarette smoking status, cigarettes per day, cigarettes per day squared, duration of smoking, duration of smoking squared, age started smoking, passive smoking, vegetable/fruit/fiber consumption, fat consumption, industrial exposures, and occupation dirtiness index
Kim et al. 2020	South Korea	Cross-sectional	28,557	58.7	NA	Alpha track detector	Medical questionnaires, physical examination, serologic tests and provided by participants	103.1 Bq/m^3^	*N*_stroke_ = 926 OR (95% CI): All: 1.242 (1.069–1.444) (*p* = 0.05) Age > 75: 1.872 (1.320–2.654) (*p*˂0.01)	Age, sex, hypertension, diabetes, dyslipidemia, ischemic heart disease, BMI, house income, education, occupation, smoking alcohol, exercise, and dietary intake

*BMI*, body mass index; *CI*, confidence interval; *CSD*, circulatory system disease; *CeVD*, cerebrovascular disease; *IHD*, ischemic heart disease; *HR*, hazard ratio; *LBL*, the Lawrence Berkeley National Laboratory; *N*, number; *NA*, not available; *OR*, odds ratio; *SMR*, standardized mortality ratio; USA, the United States of America; WLM, working-level month; Y, year

^*a*^Mean age at employment

### Quality assessment

We summarized the ROB assessment for each study in Supplemental Table 3. Overall, we judged the quality of all included studies as moderate quality (Tier 2).

### Occupational population

#### Systematic review

Among occupational studies, a recent study from French cohort with a follow-up from 1946 to 2007 of 5400 male uranium miners revealed that radon exposure was related to an increased risk of CeVD mortality (ERR/100 WLM = 0.42, 95% CI 0.04, 1.04) (Rage et al. 2018). The positive association between radon exposure and CeVD mortality was confirmed in a Chinese historic cohort (1976–1987) consisting of 17,143 tin workers (*N*_CeVD death_ = 302) (Xuan et al. 1993). When participants were separated into three groups depending on radon exposure levels (low, medium and high), there was a significant increase in CeVD mortality cross groups (RR_low_ = 1.0, RR_medium_ = 1.1, and RR_high_ = 1.3; *p* for trend < 0.001).

In contrast, studies conducted in Canada and Germany did not show a significant correlation between radon exposure and CeVD mortality. A Canadian study in Newfoundland fluorspar cohort (1950–2001), including 2070 workers (1742 underground mine workers and 328 surface mill workers, *N*_CeVD death_ = 48), found no significant association (RR = 0.49, 95% CI: 0.18, 1.34), comparing the highest (800–1600 WLM) to the lowest radon exposure level (0 WLM) (Villeneuve et al. 2007). The other Canadian study in 28,456 male miners in Ontario examined CeVD mortality in relation to radon exposure from 1950 to 2007 (*N*_CeVD death_ = 315) (Navaranjan et al. 2016), and a non-significant association was observed (ERR/100 WLM = 0.22, 95% CI − 0.12, 0.58) (Navaranjan et al. 2016). Similarly, in the most recent study from the German Wismut cohort of 58,982 male uranium miners (*N*_CeVD death_ = 2073), radon exposure was not shown to be associated with CeVD mortality during the follow-up from 1946 to 2008 (ERR/100 WLM = 0.000, 95% CI − 0.008, 0.009) (Kreuzer et al. 2013). In addition, one study pooled data from the Canadian Port Hope and German Wismut cohorts, including 6802 male workers without mining experience (*N*_stroke death_ = 252) (Zablotska et al. 2018), and found no significant association of radon exposure with stroke mortality (ERR =  − 0.07, 95% CI − 0.40, 0.52).

#### Meta-analysis

Given the conflicting results from the aforementioned occupational cohorts (Table 1), we performed a meta-analysis on four studies with available data (Kreuzer et al. 2013; Navaranjan et al. 2016; Rage et al. 2018; Zablotska et al. 2018). The final dataset comprised 99,730 male miners and/or workers without mining experience (*N*_CeVD death_ = 2745) with a follow-up duration ranging from 49 (Zablotska et al. 2018) to 62 (Kreuzer et al. 2013; Zablotska et al. 2018) years. The mean radon exposure level ranged from 10 WLM (Zablotska et al. 2018) to 280 WLM (Kreuzer et al. 2013).

**Fig. 2:**
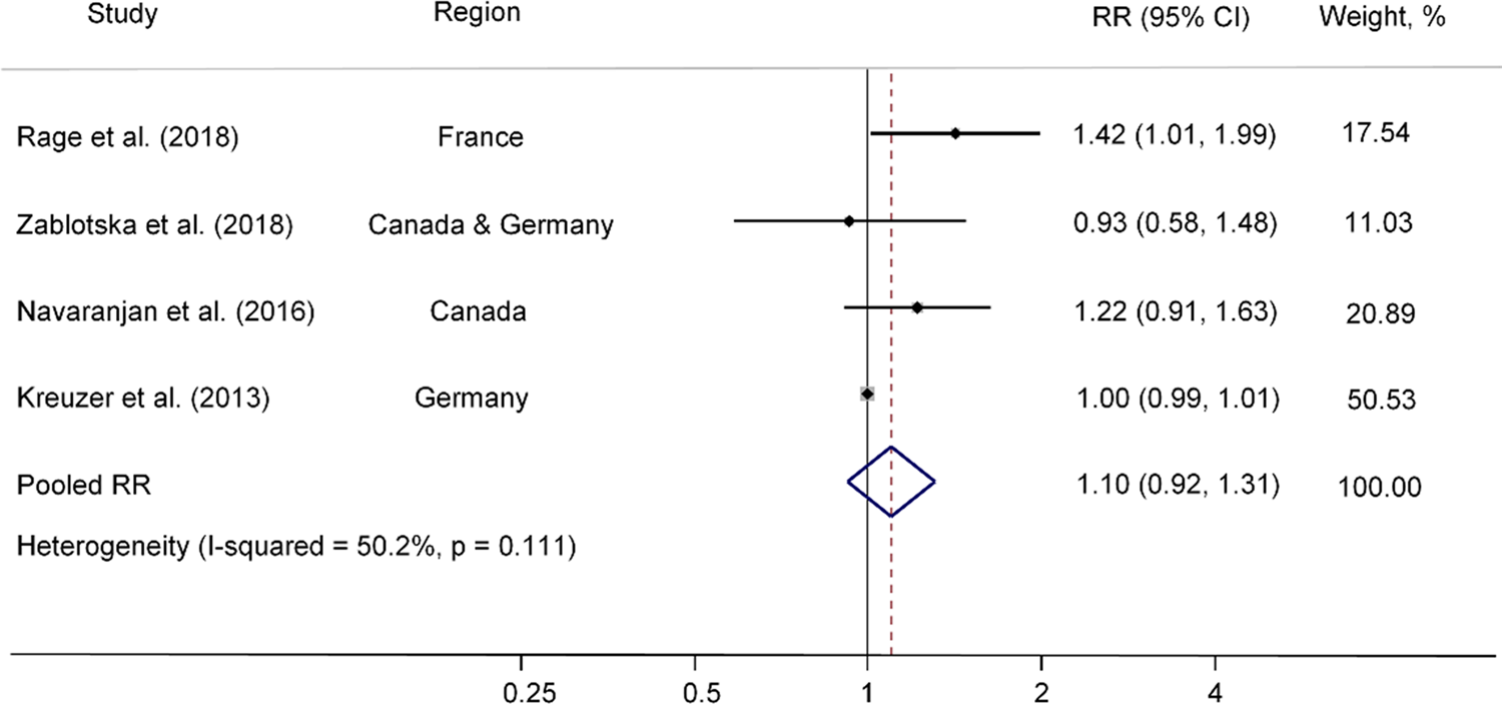
Pooled RR (95% CI) of CeVD mortality associated with radon exposure in four occupational studies. Solid dots (•) represent the RRs reported in individual studies, while the open diamonds (◇) signify the pooled RRs estimated in the meta-analysis. Horizontal lines indicate 95% CIs for the study-specific RRs

**Fig. 3:**
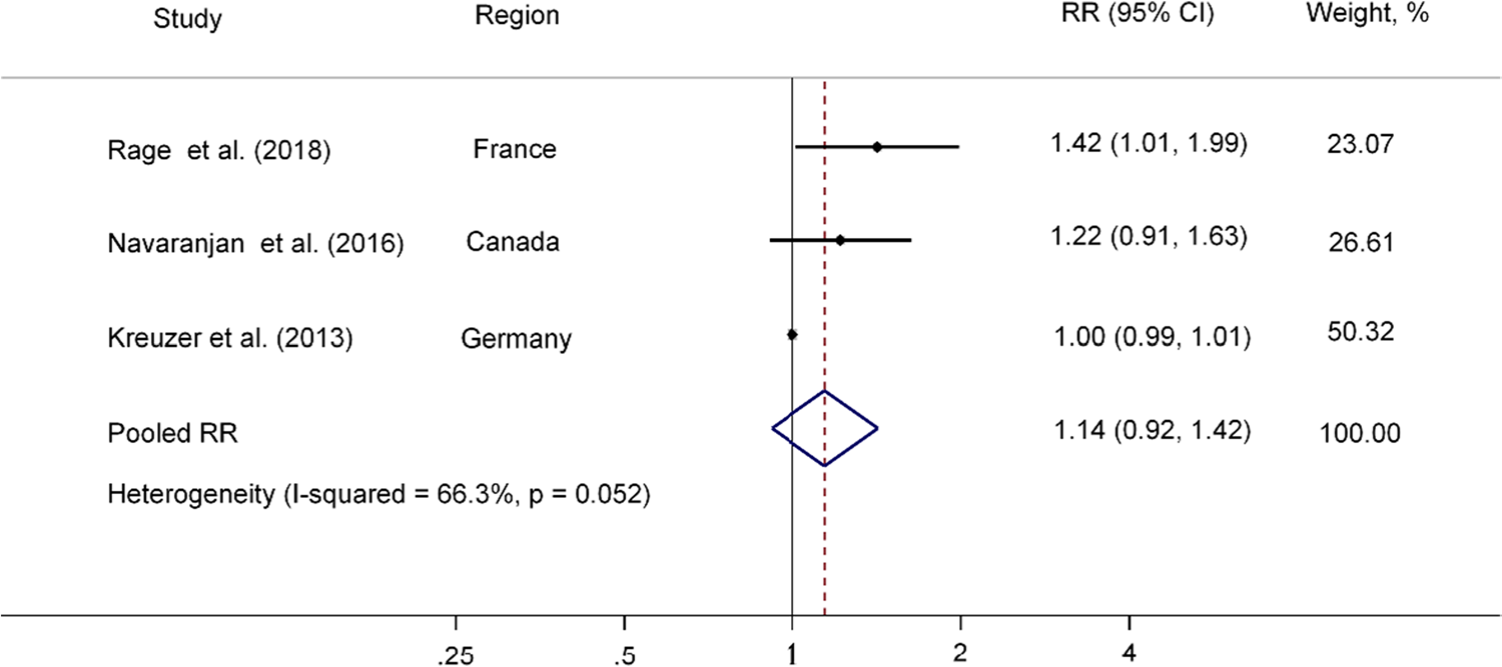
Pooled RR (95% CI) of CeVD mortality associated with radon exposure in three miner studies. Solid dots (•) represent the RRs reported in individual studies, while the open diamonds (◇) signify the pooled RRs estimated in the meta-analysis. Horizontal lines indicate 95% CIs for the study-specific RRs

The weighted RR indicated that radon exposure was not associated with CeVD mortality (Pooled RR/100 WLM = 1.10, 95% CI 0.92, 1.31; Fig. 2). The result of Egger’s test revealed no evidence of publication bias (*p* = 0.282). In the leave-one-out sensitivity analysis, no single study had a substantial influence on the pooled estimate (Supplemental Table 4). Notably, in the three cohorts comprising miners only, the pooled estimates were not substantially modified (RR: 1.14; 95% CI 0.92, 1.42; Fig. 3).

#### General population

A total of three studies were conducted in the general population with two cohort studies assessing CeVD mortality and one cross-sectional study assessing the prevalence of stroke attributed to radon. Unlike occupational studies that included primarily male workers, studies in general population consisted of both male and female individuals. The sample size ranged from 752 to 811,961. The follow-up time of the two cohort studies was 24 (Turner et al. 2012a) and 60 years (Klotz et al. 1989).

As early as 1980s, investigators recognized the potential health hazards of elevated radon exposure from industrially contaminated soil (Klotz et al. 1989). In a residential cohort of 752 individuals (*N*_CeVD death_ = 18) in New Jersey, an increased death rate for CeVD was observed as compared to the mortality of New Jersey’s general population (SMR = 1.71; 95% CI 1.01, 2.59; Table 2) (Klotz et al. 1989).

According to a nationwide cohort study (811,961 individuals, *N*_CeVD death_ = 23,344) conducted by the American Cancer Society (ACS), the association between residential radon exposure at the county-level and CeVD mortality (per 100 Bq/m^3^) was either borderline significant (HR = 1.05, 95% CI 0.99, 1.10) or significant (HR = 1.07, 95% CI 1.01, 1.14), depending on the data sources of county-level radon exposure (Table 2) (Turner et al. 2012a).

Additionally, in a nationwide cross-sectional study conducted among 28,557 (*N*_stroke death_ = 926) South Koreans, a significant increase in the prevalence of stroke was observed among residents living in houses with elevated indoor radon levels at the major city/province level (Bq/m^3^) from 2012 to winter 2016 (≤ 83.4: 1.7%; 83.5–100.7: 1.8%; 100.8–111.6: 3.0%; ≥ 111.7; 3.0%) (Kim et al. 2020). After accounting for house income, smoking, alcohol consumption, etc*.*, indoor radon exposure was reported to be positively associated with the risk of stroke (OR = 1.24, 95% CI 1.07, 1.44), especially in the elderly aged above 75 (OR = 1.87, 95% CI 1.32, 2.65) (Table 2) (Kim et al. 2020).

#### Discussion

Among the occupational population, although the existing literature included in the systematic review does not provide consistent evidence establishing a link between cumulative radon exposure and increased CeVD mortality, the pooled results of the meta-analysis from four occupational studies indicate a non-significant association of radon exposure with CeVD mortality. For the general population, published investigations consistently suggest that residential radon exposure is related to an increased CeVD risk.

While this systematic review and meta-analysis provides first-hand evidence of a potential link between radon exposure and CeVD risk, several limitations should be acknowledged. First, the outcome definition is not strictly consistent. We combined studies with CeVD or stroke as the outcome given that stroke is the most common manifestation of CeVD (Portegies et al. 2016) and the limited number of studies. Six of the included studies reported CeVD that was identified by different versions of the International Classification of Diseases (ICD) codes (e.g., ICD-8: 430–438; ICD-9: 430–438; ICD-10: I60–I69). Additionally, CeVD/stroke was reported without the ICD identification in the Korea (Kim et al. 2020) and New Jersey studies (Klotz et al. 1989), whereas the Chinese cohort identified CeVD based on the Chinese Health Ministry coding system (no code reported) (Xuan et al. 1993). Second, because of the limited number of eligible studies in the meta-analysis, we were not able to explore the impact of types of workers (i.e., underground experience yes vs*.* no) or any dose–response relationship. Third, analyses were restricted to male individuals due to the limit number of female workers in these occupational cohorts, which may pose an issue of limited generalizability. Fourth, some established risk factors or predictors for CeVD (e.g., hypertension, hypercholesterolemia, smoking, or alcohol consumption) were not adjusted in most of the studies. For example, the covariate of smoking status was adjusted in only one occupational study (Villeneuve et al. 2007) and in two general population studies (Kim et al. 2020; Turner et al. 2012a).

Given the inconsistent findings from the occupational studies, some methodological issues merit discussion. Recognized by previous studies, some biases inherited from occupational studies (Pearce et al. 2007) may attenuate any possible relation between radon exposure and CeVD risk. Since radon enters the lung first and then the blood stream via gas exchange, the dosage received by the blood and vessels is lower than that by the lung (National Research Council, 1999). Radon exposure has been shown to raise the risk of lung cancer and lung cancer mortality in miners (Al-Zoughool and Krewski 2009). The null associations observed in some occupational cohorts may be due to the competing risk of lung cancer, for example, miners may have died from lung cancer or related health conditions before the onset of CeVD. Especially among elderly individuals, the competing risk can bias the mortality estimate because of the fact that elderly population frequently suffers from multiple morbidities (Abdel-Qadir et al. 2018).

In addition, although the “heathy hire effect,” one subtype of “healthy worker effect (HWE),” resulting from healthy individuals at better chance for employment was controlled to some extent in the included studies by using the internal controls; other subtypes of HWE remained. For instance, “healthy worker survivor effect” arises when the employment duration of workers is dependent on their health status (Shah 2009). Therefore, workers who received different levels of radon exposure may have different prognostic risk for chronic diseases. Another phenomenon is the “beneficial effect of work” (Shah 2009) including the potential for more rigorous disease screening and safety intervention, which may lead to the decreased disease risk and thus attenuation of the association of interest.

Notably, cause-specific mortality was not available from several studies that reported SMR because the radon exposure could not be completely isolated from other radiation exposures such as gamma radiation, diesel exhaust, and silica dust. Also, the mortality rates of certain occupational cohorts were compared with the overall death rates of the general public; studies using external controls are in general suffering from “healthy hire bias.”

Moreover, measurement error or bias may arise when personal radon exposure was not directly measured. In most occupational cohort studies, radon measurements were retrospectively estimated based on the mean radon concentration of the mine or processing facility and individuals’ working history. Of note, the radon exposure assessment in the French cohort was more precise (Rage et al. 2018). Since 1956, individual dosimetric measurement records for radon exposure were available monthly, along with the location, period, and type of work for each miner. Post 1983, the measurement was further improved by utilizing personal dosimetry.

Another potential source of bias is the choice of lag time, the latent period between the initial exposure and the onset of disease. Assuming there is no between-individual variation in terms of the lag periods, two standard approaches were commonly used to determine a fixed lag time from a number of lag periods (e.g*.*, 5, 10, 15, and 20 years) (Richardson et al. 2011): (1) the one that maximizes the effect estimate; or (2) best goodness of fit. Among our included studies, the French cohort (Rage et al. 2018) and Chinese cohort (Xuan et al. 1993) studies selected 5 years as the lag time without accounting for other lag periods while other studies tested a variety of options although differences between models were minimal. However, all studies were based on the single lag time assumption, which may not be plausible. Some researchers proposed the likelihood-based approach which may potentially reduce the bias arising from the standard approaches.

**Fig. 4:**
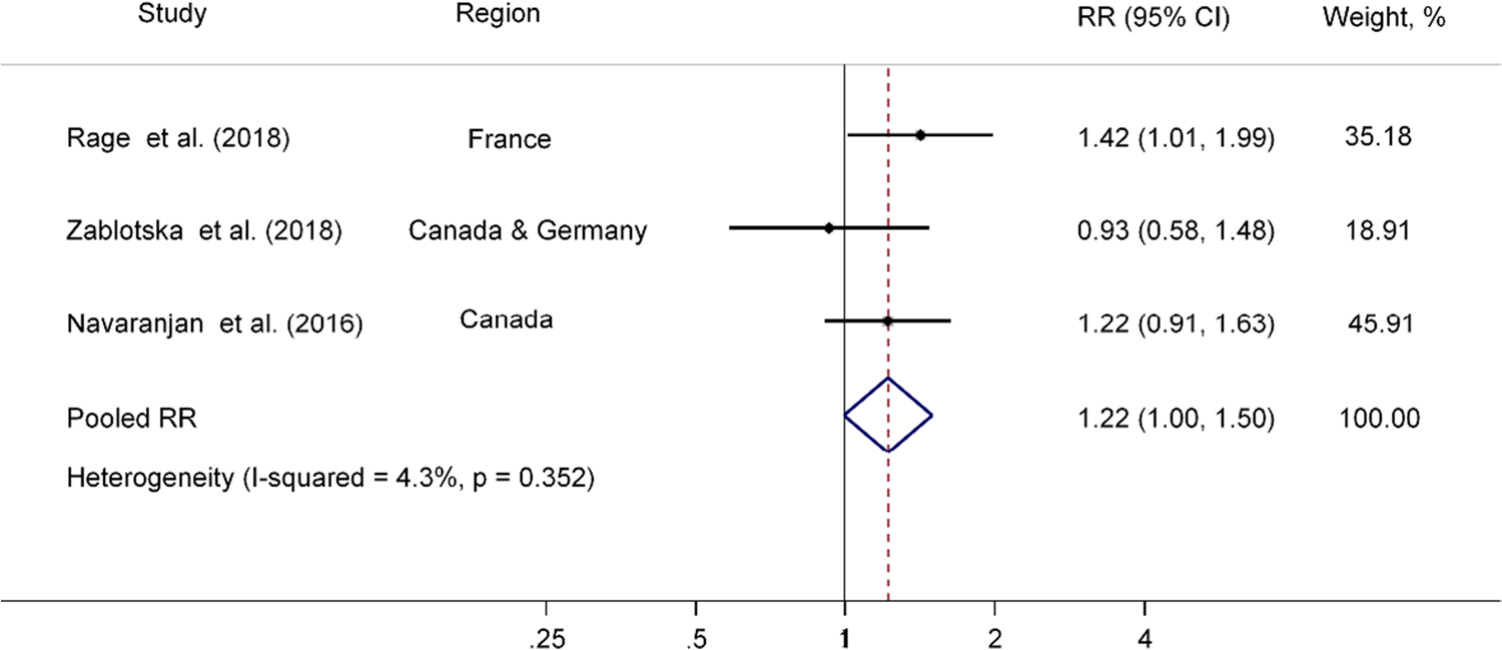
Pooled RR (95% CI) of CeVD mortality associated with similar radon exposure in three studies. Solid dots (•) represent the RRs reported in individual studies, while the open diamonds (◇) signify the pooled RRs estimated in the meta-analysis. Horizontal lines indicate 95% CIs for the study-specific RRs. CeVD, cerebrovascular disease; CI, confidence interval; RR, relative risk

In our meta-analysis, bias could also arise due to analytical issues in the calculation of 95% CI of ERR. The estimation of CIs in the ERR model is prone to errors due to measurement errors of the radon exposure (independent or correlated) (Zhang et al. 2017). The correction methods such as regression calibration and Monte Carlo method (Zhang et al. 2017) appear to be indispensable. Nevertheless, none of the included studies have reported the correction of CIs, which may have led to some uncertainties in our pooled RR. In addition, the ERR model fitted with Poisson regression assumed a linear association between exposure and disease mortality (Lee 2015). While this assumption is reasonable, a non-linear pattern or dose rate effect (Lubin et al. 1995) is also possible when the radon exposure levels are relatively high such as the values observed in the occupational studies. Among the studies included in the meta-analysis, the mean radon exposures were 10.0 WLM (Canadian and German non miners) (Zablotska et al. 2018), 21.0 WLM (Canadian miners) (Navaranjan et al. 2016), 35.1 WLM (French miners) (Rage et al. 2018), and 280 WLM (German miners) (Kreuzer et al. 2006, 2013). We therefore observed the pooled RR to be borderline significant by excluding the German-miner study, in which the mean WLM was substantially higher than that in other studies, in the sensitivity analysis (RR = 1.223, 95% CI 0.996, 1.500; Fig. 4).

A few points are worth discussion in the studies of general population. Ecologic measures of radon exposure may not be reflective of exposure estimates at the level of the individual (e.g., ecologic fallacy) especially if geographic areas are included that exhibit wide variations in radon exposure within the geographic areas (Puskin 2003). While the ACS study relied on more sophisticated measures of county-level radon exposure (Turner et al. 2012a), the ecologic assessment of indoor radon level in the South Korean study (Kim et al. 2020) was based on the average radon concentration at a major city or province level.

The study using ACS cohort reported two HRs resulting from different methods assessing the county-level ecological radon exposure (Turner et al. 2012a): methods developed by Lawrence Berkeley National Laboratory (LBL) (Price and Nero 1996) and by Cohen (Cohen 1992, 1995). Investigators reported the LBL-HRs in the main analysis (Turner et al. 2012a), partially because of a larger sample size; Cohen excluded three states, California, Arizona, and Florida, that have high population migration (Cohen 1995). We reasonably argued that Cohen-HRs should also be highlighted because the LBL-HRs may have been diluted due to the random variation introduced by those three states. Similarly, in the Medicare beneficiaries cohort study investigating the association of radon exposure at a county-level with all-cause mortality risk, whether the mortality risk was modified by patients with stroke varied by method of radon measurements (Yitshak-Sade et al. 2019). In addition, the measurement errors associated with both measures of ecological radon exposure such as seasonal/yearly variation or within-county variations may bias the association towards the null (Turner et al. 2012b).

Although research was primarily conducted in the occupational settings, we contend that residential radon should be emphasized due to the large population at risk. In studies of the general population, although the heterogeneity across studies may affect the results to some extent, the consistent findings shed some light on the risk of prolonged indoor radon exposure. Also, according to the Environmental Protection Agency (EPA) risk estimates from 1995, nearly one out of every 15 homes in the USA has radon concentration above the EPA’s action level (> 4 pCi/L) (United States Environmental Protection Agency, 2020), and there are more homes in need of radon mitigation than there were 25 years ago (Field 2012).

## Conclusion

In summary, the association of radon exposure with CeVD mortality in occupational cohorts is inconsistent, which may be explained by different methods of radon exposure assessment and other methodological issues. By contrast, although studies are limited, findings from the general population suggested that residential radon exposure is a potential risk factor for CeVD. While ecologic studies are useful for generating hypotheses, the scientific rigor of the general population studies could be substantially improved by linking individual level assessments of retrospective radon exposure with CeVD outcomes. Since radon exposure is a common public health issue, more rigorously designed epidemiologic studies, especially in the general population are warranted.

## Supplementary Information

Below is the link to the electronic supplementary material.

Supplementary file1 (DOCX 25 KB)

## Data Availability

All the STATA codes for performing the analyses and generating the results are available upon request.

## Abbreviations

ACS: American Cancer Society
CeVD: Cerebrovascular disease
CI: Confidence interval
EPA: Environmental Protection Agency
ERR: Excess relative risk
HR: Hazard ratio
HWE: Healthy worker effect
ICD: International Classification of Diseases
LBL: Lawrence Berkeley National Laboratory
N: Number
NTP/OHAT: The National Toxicology Program Office of Health Assessment and Translation
OR: Odds ratio
RDP: Radon decay products
ROB: Risk of bias
ROS: Reactive oxygen species
RR: Relative risk
SMR: Standard mortality rate
WLM: Working level months
