# Supplementary material for: Radon exposure and risk of cerebrovascular disease: a systematic review and meta-analysis in occupational and general population studies
Source: Environ Sci Pollut Res Int. 2022 Apr 23;29(30):45031–43. doi: 10.1007/s11356-022-20241-x (PMC9209369; doi:10.1007/s11356-022-20241-x)
Supplement: Supplementary file 1 — Supplementary file1 (DOCX 25 KB) [file 11356_2022_20241_MOESM1_ESM.docx]

**Radon exposure and risk of cerebrovascular disease: a systematic review and meta-analysis in occupational and general population studies**

Liping Lu^*^ • Yijia Zhang^*^ • Cheng Chen • R. William Field • Ka Kahe

**^*^** There authors contributed equally to this work

**Corresponding author**

Dr. Ka Kahe, Department of Obstetrics and Gynecology and Department of Epidemiology, Columbia University Irving Medical Center, New York 10032, USA; E-mail: kk3399@ columbia.edu.

**Journal name:** Environmental Science and Pollution Research

**Supplemental Table 1.** Details of literature search in electronic databases

| **Database** | **Search string** |
| --- | --- |
| PubMed | 1. Search: (radon) OR (RDP) Sort by: Most Recent 2. Search: (((((((cerebrovascular disease) OR (stroke)) OR (cardiovascular disease)) OR (transient ischemic attack)) OR (cerebral aneurysm)) OR (vascular malformation)) OR (vascular dementia)) OR (subarachnoid hemorrhage) Sort by: Most Recent 3. #1 AND #2 |
| Embase | 1. radon OR rdp 2. (cerebrovascular AND disease) OR stroke OR (cardiovascular AND disease) OR (transient AND ischemic AND attack) OR (cerebral AND aneurysm) OR (vascular AND malformation) OR (vascular AND dementia) OR (subarachnoid AND hemorrhage) 3. #1 AND #2 |
| Scopus | TITLE-ABS-KEY ( ( "cerebrovascular disease" OR ( stroke ) OR "cardiovascular disease" OR "transient ischemic attack" OR "cerebral aneurysm" OR "vascular malformation" OR "vascular dementia" OR "subarachnoid hemorrhage" ) AND ( ( radon ) OR ( rdp ) ) ) |
| Web of Science | 1. radon (All Fields) or RDP (All Fields) 2. (((((((ALL=(cerebrovascular disease)) OR ALL=(stroke)) OR ALL=(cardiovascular disease)) OR ALL=(transient ischemic attack)) OR ALL=(cerebral aneurysm)) OR ALL=(vascular malformation)) OR ALL=(vascular dementia)) OR ALL=(subarachnoid hemorrhage) 3. #1 AND #2 |

| **Supplemental Table 2.** PECO statement | |
| --- | --- |
| **PECO statement** |  |
| Populations | Human population studies were selected, including occupational population and general population. |
| Exposures | Exposure to radon or its decay products were included. There are no restrictions on the timing, route, level or determination of estimated exposure. |
| Comparators | Human population exposed to a lower level of radon or its decay products. |
| Outcomes | The mortality or risk of CeVD measured in the exposed population. |

Abbreviation: CeVD, cerebrovascular disease.

**Supplemental Table 3.** Summarization of risks of bias of the included studies

| **Studies** | **Selection bias** | **Confounding bias** | **Attrition bias** | **Detection bias** | | | | **Selective reporting bias** | | | **Other bias** | **Tier** |
| --- | --- | --- | --- | --- | --- | --- | --- | --- | --- | --- | --- | --- |
|  | Q1 | Q2* | Q3 | Q4* | | Q5* | | Q6 | | | Q7 |  |
| Rage et al. 2018 | Probably low | Probably high | Probably low | | Probably low | | Probably low | | Probably low | Probably low | | 2 |
| Xuan et al. 1993 | Probably low | Probably high | Probably low | | Probably low | | Probably high | | Probably low | Probably low | | 2 |
| Villeneuve et al. 2007 | Probably low | Probably high | Probably high | | Probably high | | Probably low | | Probably low | Probably low | | 2 |
| Navaranjan et al. 2016 | Probably low | Probably high | Probably low | | Probably high | | Probably low | | Probably low | Probably low | | 2 |
| Kreuzer et al. 2013 | Probably low | Probably high | Probably low | | Probably high | | Probably low | | Probably low | Probably low | | 2 |
| Zablotska et al. 2018 | Probably low | Probably high | Probably high | | Probably high | | Probably low | | Probably low | Probably low | | 2 |
| Klotz et al. 1989 | Probably low | Probably high | Probably low | | Probably low | | Probably low | | Probably high | Probably low | | 2 |
| Turner et al. 2012 | Probably low | Probably  low | Probably low | | Probably high | | Probably low | | Probably low | Probably low | | 2 |
| Kim et al. 2020 | Probably low | Probably  low | Probably low | | Probably low | | Probably high | | Probably low | Probably low | | 2 |

Footnote:

| Q1 | Did selection of study participants result in appropriate comparison groups? |
| --- | --- |
| Q2 | Did the study design or analysis account for important confounding and modifying variables? |
| Q3 | Were outcome data complete without attrition or exclusion from analysis? |
| Q4 | Can we be confident in the exposure characterization? |
| Q5 | Can we be confident in the outcome assessment? |
| Q6 | Were all measured outcomes reported? |
| Q7 | Were there no other potential threats to internal validity (*e.g*., statistical methods were appropriate and researchers adhered to the study protocol)? |
|  |  |
| Tier 1 | A study must be rated as “definitely low” or “probably low” risk of bias for key questions and have most other applicable items rated as “definitely low” or “probably low” risk of bias. |
| Tier 2 | Study does not meet the criteria for Tier 1 or Tier 3. |
| Tier 3 | A study must be rated as “definitely high” or “probably high” risk of bias for key questions and have most other applicable items rated as “definitely high” or “probably high” risk of bias. |
|  |  |
| *Key question | |
|  | |

Abbreviation: Q, question.

**Supplemental Table 4.** Leave-one-out sensitivity analysis

| **Excluded study** | **Pooled RR (95% CI)** |
| --- | --- |
| Rage et al. 2018 | 1.00 (0.99, 1.01) |
| Navaranjan et al. 2015 | 1.08 (0.86, 1.35) |
| Zablotska et al. 2018 | 1.14 (0.92, 1.42) |
| Kruezer et al. 2013 | 1.22 (0.996, 1.50) |
